# 细胞间邻近标记技术在慢性髓性白血病中的应用及其临床意义

**DOI:** 10.3760/cma.j.issn.0253-2727.2023.07.003

**Published:** 2023-07

**Authors:** 蓝蓝 艾, 安丽 赖, 小桓 覃, 兵城 刘, 劼 李, 建祥 王, 平 朱

**Affiliations:** 1 中国医学科学院血液病医院（中国医学科学院血液学研究所）、北京协和医学院，实验血液学国家重点实验室，国家血液系统疾病临床医学研究中心，细胞生态海河实验室，天津 300020 State Key Laboratory of Experimental Hematology, National Clinical Research Center for Blood Diseases, Haihe Laboratory of Cell Ecosystem, Institute of Hematology & Blood Diseases Hospital, Chinese Academy of Medical Sciences & Peking Union Medical College, Tianjin 300020, China; 2 天津医学健康研究院，天津 301600 Tianjin Institutes of Health Science, Tianjin 301600, China; 3 南京大学化学化工学院，配位化学国家重点实验室，化学和生物医药创新研究院，南京 210023 State Key Laboratory of Coordination Chemistry, Chemistry and Biomedicine Innovation Center (ChemBIC), School of Chemistry and Chemical Engineering, Nanjing University, Nanjing 210023, China

**Keywords:** 化学生物学邻近标记技术, 血液恶性肿瘤, 白血病，髓样，慢性, 肿瘤抗原反应性T细胞, 免疫表型, Biochemical proximity labeling technology, Hematological malignancy, Leukemia myeloid, chronic, Tumor antigen-specific T cell, Immunophenotype

## Abstract

**目的:**

探讨应用化学生物学邻近标记技术（Interaction-dependent fucosyl-biotinylation, FucoID），捕获血液恶性肿瘤慢性髓性白血病（CML）中肿瘤抗原特异反应性T细胞及其临床价值。

**方法:**

通过流式细胞术及荧光显微镜成像确定FucoID适用于血液肿瘤CML的实验条件。选取14例CML慢性期患者初诊样本，利用流式细胞术及FucoID对其外周血样本进行肿瘤细胞与T细胞的分离、共孵育与邻近标记，实现肿瘤抗原特异反应性T细胞的表型鉴定，并探讨FucoID在CML中的诊治价值。

**结果:**

研究首先确定了FucoID适用于血液肿瘤CML的实验条件。患者CD3^+^ T细胞比例为（8.96±6.47）％，远低于正常供者的（38.89±22.62）％。患者间肿瘤抗原特异反应性T细胞占比存在差异［（3.34±4.49）％］。CD4^+^ T细胞中肿瘤抗原特异反应性T细胞比例为（3.95±1.72）％，普遍低于CD8^+^ T细胞中肿瘤抗原特异反应性T细胞比例［（5.68±2.18）％］。相比肿瘤抗原非反应性T细胞，肿瘤抗原特异反应性T细胞中高度富集CCR7^−^ CD45RA^−^ 效应记忆性T细胞以及CCR7^−^ CD45RA^+^效应性T细胞。患者肿瘤免疫反应性强度与其外周血中WBC及HGB水平显著相关（*P*值均<0.05），与其他临床基线特征无相关性。

**结论:**

FucoID结合流式细胞术可快速鉴定及分离CML中肿瘤抗原特异反应性T细胞，该方法在CML中的成功应用提示其在血液肿瘤领域具有潜在的临床应用价值。

## Abstract (en)

**Objective:**

This study aimed to explore the application of interaction-dependent fucosyl-biotinylation（FucoID）, a chemical biology-based proximity labeling technique, in capturing tumor antigen-specific T cells and its clinical value in chronic myelogenous leukemia（CML）.

**Methods:**

Flow cytometry and fluorescence microscopy were employed to evaluate the experimental parameters for FucoID in CML. Peripheral blood samples were obtained from 14 newly diagnosed CML patients in the chronic phase. These samples underwent flow cytometry-based sorting and were subsequently labeled with FucoID to facilitate the isolation of tumor cells and T cells, followed by the immunophenotypic identification of tumor antigen-specific T cells. Finally, the diagnostic and therapeutic potential of FucoID in CML was assessed.

**Results:**

Initially, the experimental parameters for FucoID in CML were established. The proportion of CD3^+^ T cells in patients was（8.96±6.47）％, exhibiting a marked decrease compared with that in healthy individuals at（38.89±22.62）％. The proportion of tumor-specific antigen-reactive T cells was（3.34±4.49）％, which demonstrated interpatient variability. In addition, the proportion of tumor-specific antigen-active T cells in CD4^+^ T cells was（3.95±1.72）％, which was generally lower than the proportion in CD8^+^ T cells at（5.68±2.18）％. Compared with those in tumor-specific antigen-nonreactive T cells, CCR7^−^CD45RA^−^ effector memory T cells and CCR7^−^CD45RA^+^ effector T cells were highly enriched in tumor-specific antigen-reactive T cells. Moreover, the intensity of tumor immune reactivity in patients exhibited a significant correlation with white blood cell count（WBC）and hemoglobin（HGB）levels in peripheral blood, while no such correlation was observed with other clinical baseline characteristics.

**Conclusion:**

The combination of FucoID and flow cytometry enables the rapid identification and isolation of tumor antigen-specific T cells in CML. The successful application of this method in CML and the implications of our findings suggest its potential clinical value in the field of hematologic malignancies.

患者抗肿瘤免疫反应的快速精准监控对临床上疾病个性化诊疗有关键指导意义。然而，现用于鉴定分离肿瘤抗原特异反应性T细胞的策略精确度低、操作复杂、周期长，使得其基础研究与临床推广均受到一定限制[1]–[2]。化学生物学邻近标记技术（FucoID）不依赖于基因操作手段，基于细胞-细胞相互作用对特定细胞进行化学标记，首先构建细胞-酶偶联物，酶可将生物素化的底物转移到与该细胞发生相互作用的细胞表面，后续通过对生物素信号的检测可以对存在相互作用的细胞进行鉴定和分离。目前该方法已在实体肿瘤中得到成功应用，从肿瘤浸润性T细胞中区分肿瘤抗原特异反应性T细胞与旁观者T细胞，发现肿瘤抗原反应性T细胞具有独特的T细胞受体库和基因特征，此外，该方法有助于细胞间受体-配体相互作用分子对等关键信息的捕获[3]–[5]，而在血液肿瘤研究中无相关报道。因此，为了实现FucoID在血液肿瘤中的成功应用，我们将恶性克隆性疾病慢性髓性白血病（CML）作为研究对象。这是因为CML肿瘤抗原明确，BCR-ABL融合基因编码蛋白是CML关键分子特征，也是肿瘤抗原特异反应性T细胞识别肿瘤细胞的重要抗原[6]–[7]。此外，鉴定CML中肿瘤抗原特异反应性T细胞将有助于开发细胞免疫疗法，进而优化目前以酪氨酸激酶抑制剂（TKI）为主导的治疗方案，改善难治复发患者的预后[8]。本研究我们利用FucoID首次在血液恶性肿瘤CML中鉴定肿瘤抗原特异反应性T细胞，解析其免疫表型特征，并探讨其临床意义。

## 材料与方法

1. 样本来源：14例确诊为CML慢性期的初诊患者及3名健康供者外周血样本来自中国医学科学院血液病医院。该研究由中国医学科学院血液病医院伦理委员会审批（批准号：SBKT2020008-EC-2）。综合临床症状、细胞形态学、免疫表型、染色体核型、融合基因等检查结果，所有患者疾病诊断均符合WHO CML慢性期诊断标准。

2. 主要试剂：RPMI培养基、胎牛血清（FBS）、HEPES购于美国Gibco公司，PBS、Ficoll购于美国Cytiva公司，EDTA购于美国Invitrogen公司，N-乙酰-D-乳糖胺购于上海源叶生物科技有限公司，MgCl_2_购于美国Sigma-Aldrichgon公司，Hank's平衡盐溶液购于上海麦克林生化科技有限公司。

抗体CD34-FITC、CD4-Percp-Cy5.5及CellTrace™ Violet、LIVE/DEAD™ Fixable Aqua Dead Cell Stain Kit购于美国Invitrogen公司，抗体Lineage-BV510、CD3-APC-Cy7、CD8-PE-Cy7、CD45RA-PE、CCR7-FITC、Streptavidin-APC购于美国BioLegend公司。

配制PBE（PBS+2％FBS+2 mmol/L EDTA）、Fucosylation（HBSS+3 mmol/L HEPES+20 mmol/L MgCl_2_+0.5％FBS）、FACS（DPBS+2％FBS+1.5 mmol/L EDTA）、Co-culture（RPMI 1640+10％FBS+20 mmol/L MgCl_2_）缓冲液用于细胞处理与FucoID邻近标记。

化学试剂GDP-fucose-conjugated H. pylori α（1,3）Fucosyltransferase（GDP-Fuc-FT），GDP-fucose-conjugated Biotin（GDP-Fuc-Biotin）由南京大学李劼教授课题组惠赠。

3. 细胞分选：复苏且由氯化铵溶血素裂解红细胞后的样本重悬于100 µl PBE缓冲液中，按照抗体说明书推荐用量加入抗体：20 µl Lineage-BV510、5 µl CD34-FITC、5 µl CD3-APC-Cy7，冰上避光孵育30 min。用PBE缓冲液洗涤重悬至1 ml，加入1 µl DAPI后用流式分选仪检测。将Lineage^−^ CD34^+^白血病原始细胞、CD3^+^T细胞分选至PBE缓冲液中用于后续FucoID邻近标记。

4. 细胞相互作用荧光显微镜成像：将CML患者外周血原始细胞（即Blast细胞）与T细胞以20 000∶20 000细胞量等比混匀至100 µl Co-culture缓冲液，细胞密度为4×10^5^/ml，共孵育至96孔板后，置于孵箱（37 °C，21％ O_2_，5.5％ CO_2_）。分别选取共孵育时间点0.5、1及2 h采用Leica倒置荧光显微镜明视场直接对孔内细胞间相互作用形态进行成像采集。

5. FucoID细胞间邻近标记：将Blast细胞重悬至100 µl Fucosylation缓冲液中，加入GDP-Fuc-FT（终浓度0.2 mg/ml），冰上标记30 min。将T细胞重悬至1 ml FACS缓冲液中，加入1 µl BV421-细胞示踪荧光探针，室温标记20 min，加入500 µl FBS置于冰上淬灭5 min。将洗净后的Blast细胞与T细胞以20 000∶20 000细胞量等比混匀至100 µl Co-culture 缓冲液中，细胞密度为4×10^5^/ml，细胞混匀至孔板后，置于孵箱孵育2 h（37 °C，21％ O_2_，5.5％ CO_2_），缓慢加入GDP-Fuc-Biotin（终浓度50 µmol/L），置于孵箱继续孵育0.5 h。随后加入LacNAc（终浓度2.5 mmol/L）淬灭20 min，FACS缓冲液洗净。

6. 肿瘤抗原特异反应性T细胞鉴定与免疫表型检测：共孵育孔中细胞分为两个体系分别加入抗体①Streptavidin-APC、alive-BV510、CD4-Percp-Cy5.5、CD8-PE-Cy7；②Streptavidin-APC、alive-BV510、CD45RA-PE、CCR7-FITC，分别进行CD4/CD8以及CCR7/CD45RA免疫表型的检测。标记体系为20 µl，各抗体用量按照抗体产品说明书推荐用量1∶50稀释，均为0.1 µl/孔。冰上避光孵育30 min，PBE缓冲液清洗，重悬后，采用BD FACSAriaⅢ流式细胞分选仪进行检测。

7. 统计学处理：采用SciPy v1.10.1（scipy.stats）软件进行数据分析，根据数据分布类型，正态分布资料采用均数±标准差表示，采用*t*检验。分类变量资料采用百分率表示，组间比较采用Fisher检验。*P*<0.05为差异具有统计学意义。

## 结果

一、基于FucoID成功鉴定CML肿瘤抗原特异反应性T细胞

1. CML肿瘤细胞与淋巴细胞占比：我们探究CML患者外周血单个核细胞中T淋巴细胞与肿瘤细胞的占比，并以此作为FucoID细胞共孵育比例的参考依据。患者CD3^+^T细胞比例为（8.96±6.47）％，低于正常对照组的（38.89±22.62）％，而CD34^+^Blast细胞比例为（5.95±6.47）％，高于正常对照组的（0.06±0.06）％。患者CD34^+^T细胞/CD3^+^T细胞比例约1.2∶1。结合以往研究[3],[9]，捕获CML反应性T细胞的FucoID共孵育比例定为1∶1。

2. 细胞-细胞相互作用成像：将肿瘤细胞与T细胞1∶1混匀至孔板中，我们选取共孵育时间0.5、1、2 h，对细胞-细胞相互作用进行免疫荧光成像。结果表明在体外共孵育0.5 h时细胞分布均匀；共孵育1 h时肿瘤细胞与T细胞相互靠近成簇；共孵育2 h时细胞相互作用更紧密，形成细胞团块（图1）。因此FucoID捕获CML肿瘤-免疫相互作用合适的时间点为共孵育后2 h。

**图1:**
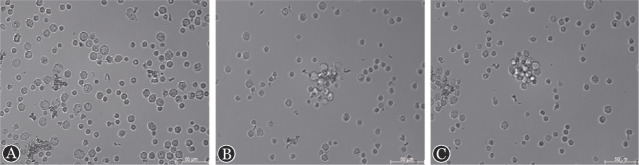
细胞-细胞相互作用动态变化 A 肿瘤细胞与T细胞共孵育0.5 h成像；B 肿瘤细胞与T细胞共孵育1 h成像；C 肿瘤细胞与T细胞共孵育2 h成像

3. 基于FucoID邻近标记成功捕获肿瘤抗原特异反应性T细胞：将CML肿瘤细胞与GDP-Fuc-FT化学复合物孵育，构建GDP-Fuc-FT-细胞偶联物。将肿瘤细胞与T细胞1∶1混合，共孵育时间为2 h，加入游离的反应底物GDP-Fuc-Biotin，肿瘤细胞表面GDP-Fuc-FT转移酶将游离GDP-Fuc-Biotin转移到与其发生相互作用的T细胞表面，形成GDP-Fuc-Biotin-细胞偶联物，实现肿瘤抗原特异反应性T细胞的Biotin标记。我们将未进行GDP-Fuc-FT偶联处理的肿瘤细胞与T细胞进行共孵育，其他实验条件与实验组保持一致，将该条件下T细胞的Biotin信号作为阴性对照。经FucoID邻近标记，14例患者的肿瘤抗原特异反应性T细胞的比例不一［（3.34±4.49）％］，我们将样本分为强肿瘤免疫反应性（strong immunoreactivity，SI）组（4例，Biotin^+^细胞比例>2％）和弱肿瘤免疫反应性（weak immunoreactivity，WI）组（10例，Biotin^+^细胞比例≤2％）。以例1为例，我们对T细胞进行免疫抗体标记，区分CD4^+^T细胞与CD8^+^T细胞，分别检测其Biotin信号，发现6.61％的CD4^+^T细胞、7.31％的CD8^+^T细胞呈Biotin阳性，该群细胞被定义为肿瘤抗原特异反应性T细胞（图2）。因此，基于FucoID邻近标记可成功对CML中肿瘤抗原特异反应性T细胞进行捕获，CD4^+^T细胞及CD8^+^T细胞共同参与CML抗肿瘤免疫反应。

**图2:**
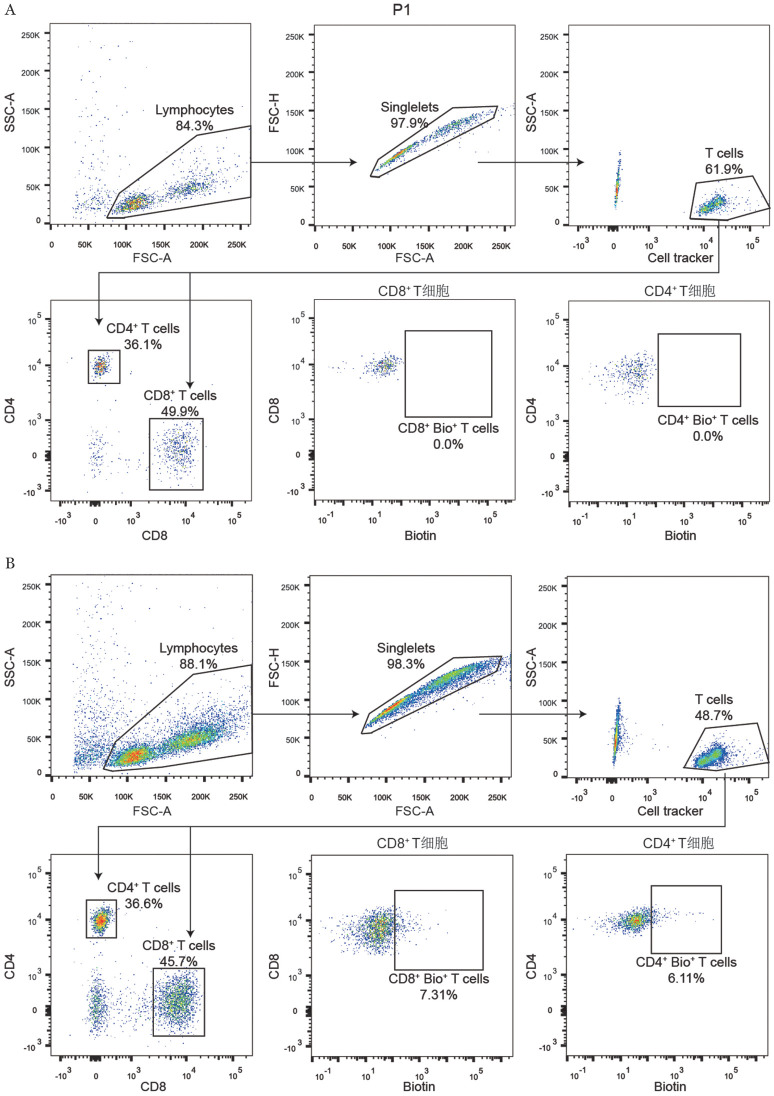
基于FucoID邻近标记捕获慢性髓性白血病肿瘤抗原特异反应性T细胞 A 流式细胞术检测T细胞Biotin信号阴性对照组；B 流式细胞术检测T细胞Biotin信号实验组

二、肿瘤抗原特异反应性T细胞免疫表型

1. CML肿瘤抗原特异反应性T细胞CD4/CD8免疫表型：通过对14例患者CD4^+^T细胞及CD8^+^T细胞中肿瘤抗原特异反应性T细胞占比情况进行统计，我们发现其中11例患者中，CD8^+^T细胞中肿瘤抗原特异反应性T细胞占比为（5.68±2.18）％，均高于CD4^+^T细胞中肿瘤抗原特异反应性T细胞占比的（3.95±1.72）％，图3为其中3例患者（例2～4）流式细胞术结果。该结果提示CD8^+^细胞在CML抗肿瘤免疫中发挥更重要的直接效应功能。

**图3:**
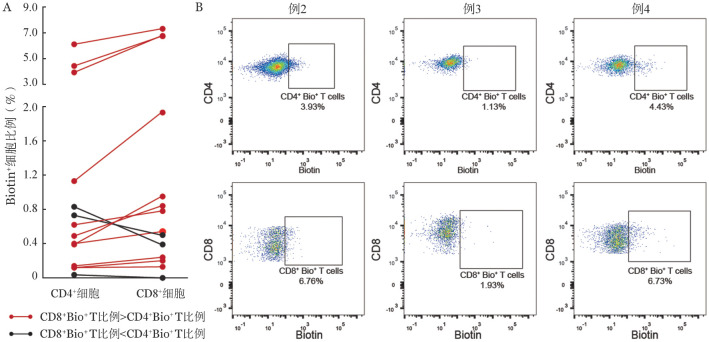
慢性髓性白血病患者CD4^+^及CD8^+^T细胞内肿瘤抗原特异反应性T细胞占比 A 14例患者来源肿瘤抗原反应性T细胞在CD4^+^及CD8^+^T细胞中的比例；B 3例患者肿瘤抗原反应性T细胞比例

2. CML肿瘤抗原特异反应性T细胞活化状态：我们基于趋化因子受体7（CCR7）和CD45RA的表达对Biotin^+^T细胞进一步区分为幼稚T细胞（CCR7^+^CD45RA^+^）、中央记忆性T细胞（CCR7^+^CD45RA^−^）、效应记忆性T细胞（CCR7^−^CD45RA^−^）以及终末分化效应性T细胞（CCR7^−^CD45RA^+^），分别检测Biotin^−^T细胞以及Biotin^+^T细胞中功能亚群组分。相比于Biotin^−^ T细胞，Biotin^+^T细胞富集CCR7^−^CD45RA^−^效应记忆性T细胞以及CCR7^−^ CD45RA^+^效应性T细胞，表明其抗肿瘤活性，反之，Biotin^−^ T细胞高度富集幼稚T细胞（图4），且该组分差异具有统计学意义（*P*<0.05）。

**图4:**
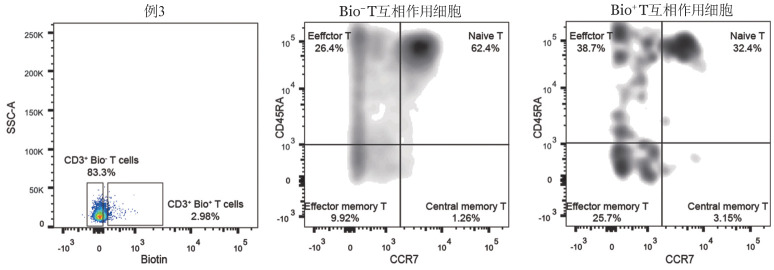
肿瘤抗原特异反应性T细胞及肿瘤抗原非反应性T细胞活化状态

三、利用FucoID评估患者肿瘤细胞免疫反应性的临床意义

根据患者肿瘤抗原特异反应性T细胞的比例将14例患者分为SI组和WI组。SI组的年龄、PLT水平、外周血原始细胞比例、外周血嗜碱性粒细胞比例、活性网状纤维、脾脏距肋缘最大距离等临床特征与WI组相比差异均无统计学意义（*P*值均>0.05），SI组的外周血WBC、HGB与WI组相比差异均有统计学意义（*P*值均<0.05）（表1）。

**表1:** 慢性髓性白血病强肿瘤免疫反应性（SI）组与弱肿瘤免疫反应性（WI）组基线临床特征比较

组别	例数	年龄（岁）	WBC（×10^9^/L）	HGB（×10^9^/L）	PLT（×10^9^/L）	外周血原始细胞（%）	外周血嗜碱性粒细胞（%）	活性网状纤维	脾脏（cm）^a^
SI组	4	32.5 ±13.8	330.9 ±189.1	99.3 ±10.1	319.3 ±150.8	3.8 ±1.8	8.8 ±4.0	1.8 ±0.4	9.8 ±6.4
WI组	8	39.7 ±10.5	103.1 ±50.1	130.7 ±11.6	398.7 ±197.4	1.8 ±2.3	6.9 ±3.5	1.1 ±0.6	3.8 ±3.8

*P*值		0.348	0.007	0.001	0.515	0.195	0.440	0.160	0.094

**注** ^a^距肋缘最大距离（cm）

## 讨论

肿瘤抗原特异反应性T细胞的鉴定是患者肿瘤免疫动态监测与个性化细胞免疫疗法开发的关键。然而，鉴定肿瘤抗原特异反应性T细胞的大多数方法具有一定局限性，如精准度不高，耗时耗力等[1]–[2]，限制了该类方法的临床应用。美国加州Scripps研究所的吴鹏教授及南京大学的李劼教授团队开发了一种细胞相互作用捕获体系FucoID[3],[5]，利用该方法可快速检测肿瘤内源性抗原特异性T细胞。该方法基于细胞-细胞相互作用对细胞进行标记，首先构建细胞-FT转移酶偶联物，FT转移酶可将生物素化的底物转移到与该细胞发生相互作用的细胞表面，后续通过对Biotin信号的检测可以对存在相互作用的细胞进行鉴定和分离。该方法已成功应用于实体肿瘤，对肿瘤抗原特异反应性T细胞进行鉴定及相互作用分子对解析[3]–[5]，具有广阔临床应用前景。然而目前该方法在血液系统恶性肿瘤中还未得到应用。

以往研究表明，在CML患者中，抗肿瘤免疫可控制介导疾病复发与进展的白血病干细胞，维持长期免疫应答，进而达到可持续的缓解[10]–[11]，因此免疫疗法在CML治疗中的应用可能会大大改善患者的预后[8],[12]。此外，对患者肿瘤免疫状态进行实时监测可为个性化诊疗方案提供科学依据。在本研究中，我们首次将FucoID应用于血液肿瘤中，成功对CML患者初诊时期的肿瘤抗原特异反应性T细胞进行捕获与免疫表型鉴定。

CML患者与健康供者相比，CD34^+^Blast细胞与T细胞占比均存在显著差异，患者T细胞占比低于健康对照，该现象与以往研究结果一致，认为CML患者机体存在细胞免疫紊乱[12]，因此从中鉴定功能激活性的肿瘤抗原特异反应性T细胞对于CML肿瘤免疫微环境的研究是十分有必要的。首先，我们探索FucoID适用于血液肿瘤CML的最佳实验条件。其一，通过统计患者CD34^+^/CD3^+^细胞比值并结合以往研究，FucoID捕获CML相互作用细胞的共孵育比例为1∶1。其二，共孵育时间是FucoID成功捕获肿瘤抗原特异反应性T细胞的另一关键要素，我们通过显微荧光成像发现，在共孵育时间达到2 h时，细胞间相互作用稳定，是捕获相互作用的最佳时间。

我们对14例患者外周血样本进行Blast细胞与T细胞的分离和FucoID邻近标记，捕获与肿瘤细胞存在相互作用的T细胞——肿瘤抗原特异反应性T细胞。患者间肿瘤抗原特异反应性T细胞的比例不一，提示抗肿瘤免疫水平具有个体异质性，后续我们根据肿瘤抗原特异反应性T细胞比例将患者分为SI组和WI组。与以往研究一致，CD4^+^T细胞与CD8^+^T细胞共同参与肿瘤杀伤作用[13]–[14]，其中CD8^+^T细胞在CML抗肿瘤免疫中发挥更重要的直接效应功能。此外，相比于幼稚T细胞在非反应性T细胞中富集，效应记忆性T细胞与效应T细胞在肿瘤抗原特异反应性T细胞中富集，证明与肿瘤细胞发生相互作用的T细胞处于活化状态。然而仍有部分反应性T细胞处于幼稚状态，针对这一现象我们做两方面猜测：首先，与以往研究一致，干性T细胞同样具有抗肿瘤效应，而且在治疗过程中可以对肿瘤细胞发挥长久的杀伤效应[15]；其次，在捕获细胞-细胞相互作用时，该方法不可避免地引入一部分“噪音”。

为了进一步探究FucoID的临床意义，我们对患者抗肿瘤免疫强度与临床基线特征进行相关性分析。值得注意的是，SI组患者的WBC、HGB与WI组患者相比差异均有统计学意义（*P*<0.05）。已有多项研究报道，CML慢性期患者初诊时期WBC、HGB等参数可以用于预测TKI治疗的分子学反应[16]–[17]，高WBC、低HGB水平与较低的深层分子反应率显著相关。结合我们的研究结果，相比低WBC、高HGB水平的患者，高WBC、低HGB水平的患者在初诊时期T细胞介导的抗肿瘤效应更强，推测当T细胞处于更顽固的肿瘤免疫微环境时，其抗肿瘤效应更活跃。因此我们认为，该类患者可以考虑在初次治疗时结合T细胞介导的免疫疗法使得患者达到更深层的分子反应。因此本研究中，采用FucoID快速鉴定并分离肿瘤抗原反应性T细胞，有助于加深对肿瘤免疫微环境的生物学理解，进而推动个性化免疫疗法的开发。

本次研究不足之处在于入组患者较少，且治疗疗程较短，无TKI治疗反应等信息，因此无法探究初诊时期抗肿瘤免疫状态与TKI治疗疗效的相关性。且本次研究采样为单一时间点，未进行患者治疗过程中抗肿瘤免疫反应的多点监测。因此我们后续计划纳入更多临床样本开展更深入的研究，将FucoID的应用与临床治疗充分结合，为CML诊治提供更为丰富的信息。

综上所述，FucoID检测肿瘤抗原特异反应性T细胞精确、可靠、快速，本研究将该方法成功应用于CML。研究结果表明，CML肿瘤微环境中存在免疫紊乱，且具有一定水平的抗肿瘤免疫反应，该反应强度具有个体异质性，具体体现为肿瘤抗原特异反应性T细胞比例不一。通过免疫表型鉴定，活化状态的CD8^+^T细胞在CML抗肿瘤反应中起关键作用。此外，患者肿瘤免疫强度与关键临床指征显著相关。这些数据表明，旨在增强抗肿瘤免疫反应的细胞疗法可能进一步改善CML患者预后。FucoID方法在CML中的成功应用提示其在血液系统恶性肿瘤诊治中的临床意义及巨大应用前景。
